# Synthesis of Polymer-Lipid Nanoparticles by Microfluidic Focusing for siRNA Delivery

**DOI:** 10.3390/molecules21101314

**Published:** 2016-10-17

**Authors:** Yujing Li, Xueqin Huang, Robert J. Lee, Yuhang Qi, Kaikai Wang, Fei Hao, Yu Zhang, Jiahui Lu, Qingfan Meng, Shuai Li, Jing Xie, Lesheng Teng

**Affiliations:** 1 School of Life Sciences, Jilin University, Qianjin Street No. 2699, Changchun 130012, China; liyujing_jlu@126.com (Y.L.); M13843046552@163.com (X.H.); lee.1339@osu.edu (R.J.L.); qi13596940673@163.com (Y.Q.); 18844545746@163.com (K.W.); haofei123789@126.com (F.H.); steadyyangguang@163.com (Y.Z.); lujh@jlu.edu.cn (J.L.); mengqf@jlu.edu.cn (Q.M.); ls2012@jlu.edu.cn (S.L.); 2 Department of Chemistry and Pharmacy, Zhuhai College of Jilin University, Zhuhai 519041, China; 3 Division of Pharmaceutics, College of Pharmacy, The Ohio State University, Columbus, OH 43210, USA

**Keywords:** microfluidic, polymer, lipid nanoparticles, siRNA

## Abstract

Polyethylenimine (PEI) as a cationic polymer is commonly used as a carrier for gene delivery. PEI-800 is less toxic than PEI-25K but it is also less efficient. A novel nanocarrier was developed by combining PEI-800 with a pH-sensitive lipid to form polymer-lipid hybrid nanoparticles (P/LNPs). They were synthesized by microfluidic focusing (MF). Two microfluidic devices were used to synthesize P/LNPs loaded with VEGF siRNA. A series of P/LNPs with different particle sizes and distributions were obtained by altering the flow rate and geometry of microfluidic chips, and introducing sonication. Furthermore, the P/LNPs can be loaded with VEGF siRNA efficiently and were stable in serum for 12 h. Finally, P/LNPs produced by the microfluidic chip showed greater cellular uptake as well as down-regulation of VEGF protein level in both A549 and MCF-7 with reduced cellular toxicity. All in all, the P/LNPs produced by MF method were shown to be a safe and efficient carrier for VEGF siRNA, with potential application for siRNA therapeutics.

## 1. Introduction

Vascular endothelial cell growth factor (VEGF) is a growth factor associated with neovascularization, which can serve as a target for cancer therapy [1]. Small interfering RNA (siRNA) targeting VEGF has attracted increasing interest as a promising strategy [2]. However, successful siRNA clinical application relies on safe and efficient vector systems. Considering immunogenic/inflammatory responses that can be induced by viral vectors, many non-viral delivery systems have been developed [3,4]. Cationic nanoparticles are the most promising among these systems [5,6]. 

Polyethylenimine (PEI) is a well-known delivery agent for nucleic acids [7,8]. However, cytotoxicity has been a significant issue for its application. PEI with a molecular size of 800 is less toxic but also less efficient when compared with the high molecular weight of PEI. Modifications of PEI and its combination with other materials have been reported to enhance the efficiency while reducing toxicity [9,10,11].

Microfluidic focusing (MF) is a novel technique for producing nanoparticles [12,13]. Compared with the conventional bulk-mixing (BM) method, MF method can be precisely manipulated and controlled, yielding homogeneous nanoparticles [14,15,16,17,18,19]. As a result, some processing steps such as extrusion and high pressure homogeneration used after the BM method are not required, thus simplifying overall nanoparticle synthesis.

Polymer-lipid hybrid nanoparticles possess both the characteristics of liposomes and nanoparticles, such as high stability, biocompatibility and controlled release properties, which means this type of nanoparticles has great promise in drug delivery applications [20,21]. To create a safe and efficient carrier, we combined PEI-800 with pH-sensitive lipid (1,2-Dioleyloxy-*N*,*N*-dimethyl-3-aminopropane, DODMA) and other helper lipids to form polymer-lipid hybrid nanoparticles (P/LNPs) via microfluidc focusing synthesis. First, we explored optimization of the parameters on P/LNPs synthesis using two microfluidic chips. These two chips are different in geometry and are shown in the Graphic Abstract. We performed a series of experiments to evaluate their transfection efficiency and cytotoxicity, using P/LNPs produced by BM method as a control. The results showed that P/LNPs containing VEGF siRNA produced by MF method can provide more desirable physicochemical and siRNA delivery properties in vitro compared to those by BM system.

## 2. Results

### 2.1. The Effect of Flow Rate and Sonication on Synthesis of P/LNPs

**Figure 1:**
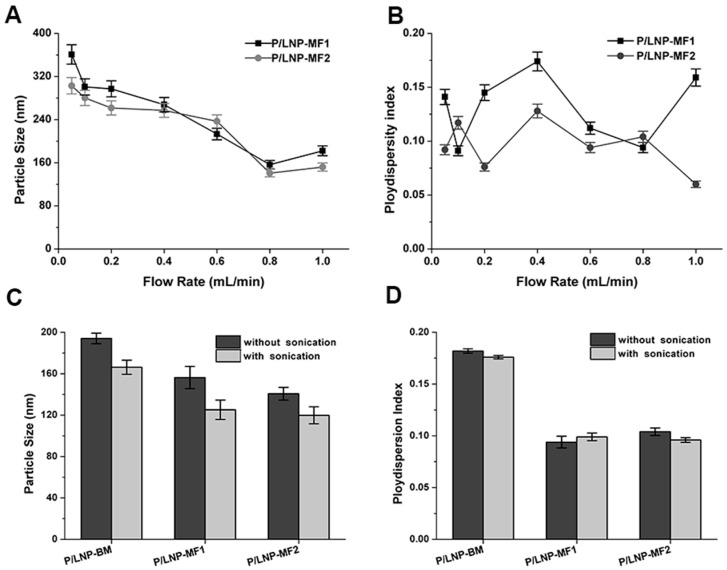
Effects of flow rate and sonication on synthesis of polymer-lipid hybrid nanoparticles (P/LNPs). (**A**,**B**) Influence of flow rate on particle size and polydispersity index; (**C**,**D**) Influence of the sonication on particle size and polydispersity index. Error bars indicate standard deviations. (*n* = 3).

In order to investigate the influence of flow rate on particle size of P/LNPs, a syringe pump was used to tune flow rate precisely. Particle size and polydispersity index were determined by dynamic light scattering and results are shown in Figure 1. With the increase of flow rate from 0.1 mL/min to 1 mL/min, the mean particle size of P/LNPs-MF1 remarkably decreased from 370 nm to 110 nm except for a slight increase during flow rate change from 0.8 mL/min to 1 mL/min, and a similar trend was also observed in the P/LNPs-MF2. Moreover, the values of mean particle size for P/LNPs-MF1 were slightly larger than those of P/LNPs-MF2 (Figure 1A), which indicated that the MF-2 chip with s-type channel has a higher mixing performance due to enlarged diffusion area.

To determine the sonication effect, we fixed the flow rate at 0.8 mL/min and synthesized the P/LNPs with or without sonication, and the sonication was performed immediately after the MF method. The result showed that sonication significantly reduced the particle size of P/LNPs, but had little impact on size distribution (Figure 1C,D). Interestingly, the average particle size of P/LNPs-BM synthesized with sonication was similar to the P/LNPs-MF1 synthesized without sonication, which indicated that the MF method can produce smaller P/LNPs with a narrower distribution. Furthermore, P/LNPs-MF2 was smaller in size than corresponding P/LNPs-MF1 with or without sonication, revealing that MF2 had a better mixing efficiency when compared with MF1. However, as shown in Figure 1D, little difference was observed in size distributions of P/LNPs regardless of the use of sonication, indicating that sonication might not work for narrowing down the particle distribution.

### 2.2. Gel Retardation Assay

**Figure 2:**
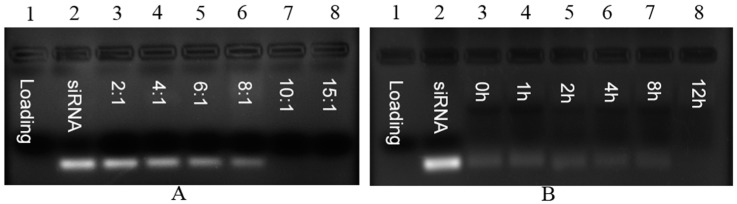
Gel retardation assay. (**A**) Electrophoretic analysis of P/LNPs-small interfering RNA (siRNA) at various mass ratios; (**B**) Stability study of P/LNPs-siRNA in 50% serum.

In order to evaluate the binding between siRNA and P/LNPs, a gel retardation assay was carried out. siRNA loaded P/LNPs were prepared at different mass ratios and loaded onto the gel. In Figure 2A, the disappearance of siRNA band in the gel indicates that binding occurred between siRNA and P/LNPs, and siRNA binding was observed at the mass ratio of 10:1, suggesting that siRNA was completely compacted into P/LNPs. In a serum stability study (Figure 2B), siRNA loaded P/LNPs at the mass ratio of 10:1 was incubated with 50% serum for 0, 1, 2, 4, 8, 12 h. No siRNA bands were observed at these time points, demonstrating that these P/LNPs had strong siRNA affinity and remain stable in serum for 12 h.

### 2.3. Cytotoxicity Study

**Figure 3:**
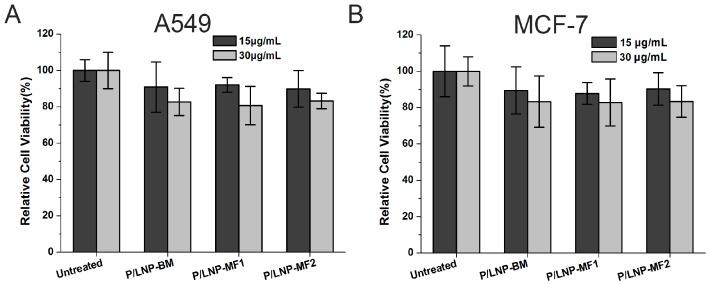
Cytotoxicity studies of P/LNPs using MTT assay on A549 cells and MCF-7 cells. Cells were treated at the transfection concentration (15 μg/mL) and double transfection concentration (30 μg/mL) for 48 h, cell viability was measured by MTT assay. (**A**) A549 cells; (**B**) MCF-7 cells. The data are displayed as mean ± SD (*n* = 3).

The cytotoxicity of P/LNPs on MCF-7 cells and A549 cells were assessed by MTT assay. In Figure 3, no sign of toxicity was observed in either of the two cells at the concentration used for transfection (15 μg/mL). Even after doubling concentrations used for transfection (30 μg/mL) for 48 h, the cell viability was still at over 80% across all P/LNPs groups, which strongly validated that this PEI-based P/LNPs formulation was safe for siRNA to deliver in vitro.

### 2.4. Cellular Uptake by Flow Cytometry

**Figure 4:**
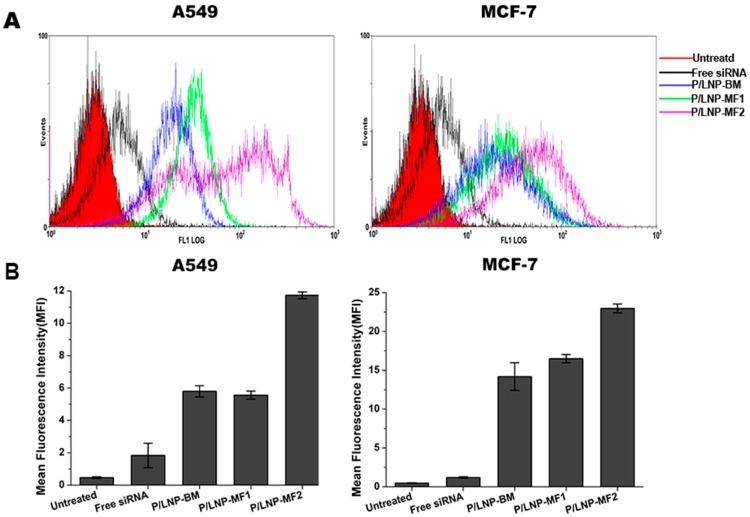
Uptake of FAM-siRNA loaded P/LNPs in A549 cells and MCF-7 cells by flow cytometry. Cells were incubated with different P/LNPs at 37 °C for 4 h, and then analyzed by flow cytometry. (**A**) Fluorescence signals intensity graphs; (**B**) the mean fluorescence intensities of A549 cells and MCF-7 cells. The data are displayed as mean ± SD (*n* = 3).

Efficient intracellular delivery of siRNA plays a critical role in the RNA interference process. Here, we used a Beckman Coulter EPICS XL flow cytometry to analyze the cellular uptake of FAM-labeled P/LNPs by MCF-7 and A549 cells and the results are shown in Figure 4. As expected, fluorescence peaks of cells treated with P/LNPs moved to the right direction when compared to those of cells treated with free siRNA, demonstrating FAM-siRNA has been delivered into the cells. In addition, the fluorescence intensity of the cells treated with P/LNPs-MF2 was found to be the highest among all groups in both MCF-7 cells and A549 cells. Taken together, P/LNPs-MF2 is shown to strongly improve cellular uptake.

### 2.5. Confocal Microscopy

**Figure 5:**
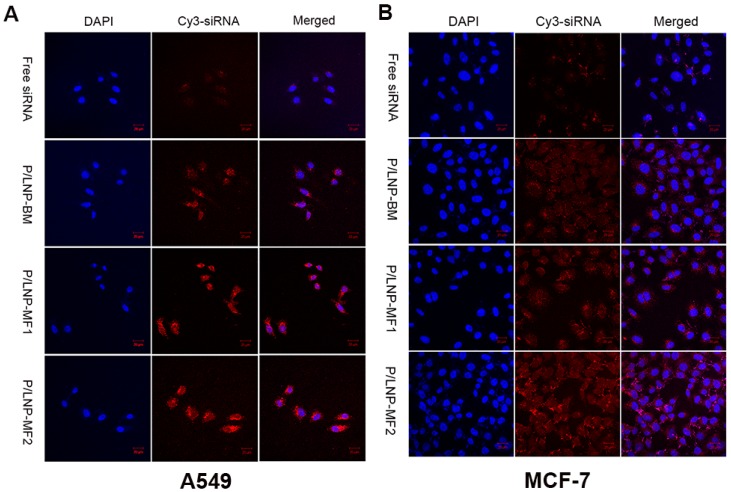
Confocal microscopy images of intracellular trafficking of (Cy3-siRNA loaded P/LNPs. (**A**) A549 cells; (**B**) MCF-7 cells. Cy3-siRNA is shown by red fluorescence, and DAPI(4′-6-diamidino-2-phenylindole) stained nuclei is shown by blue fluorescence.

In order to visualize uptake of P/LNPs by MCF-7 cells and A549 cells, confocal microscopy was employed. Cy3-labeled siRNA (Cy3-siRNA) was loaded into P/LNPs and DAPI was used to stain the nuclei. As shown in Figure 5, the labeled siRNA was observed in the cells and cells treated with P/LNPs had higher fluorescence intensity than those treated with free siRNA. Moreover, the highest fluorescence intensity was observed in the cells treated with P/LNPs-MF2. In other words, the P/LNPs-MF2 showed greater internalization than P/LNPs-MF1 and P/LNPs-BM which is consistent with the flow cytometry results.

### 2.6. Determination of VEGF Protein Expression by Western Blot

**Figure 6:**
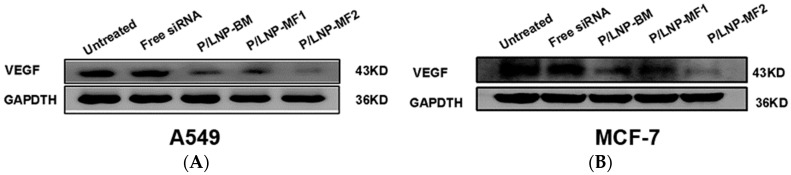
The expression of vascular endothelial cell growth factor (VEGF) in protein levels evaluated by Western blot. (**A**) A549 cells; (**B**) MFC-7 cells.

In order to determine whether siRNA was delivered by P/LNPs efficiently into the cells, the levels of VEGF proteins expression were measured by Western blot at 48 h post-transfection. As shown in Figure 6, VEGF expression in both A549 and MCF-7 cells were significantly knocked down by siRNA loaded P/LNPs and siRNA loaded P/LNPs-MF2 induced more down-regulation of VEGF protein than P/LNPs-BM and P/LNPs-MF1. These findings are consistent with the results of transfection efficiency experiments and they clearly demonstrate that P/LNPs-MF2 can efficiently deliver VEGF siRNA into tumor cells and consequently induce down-regulation of VEGF protein.

## 3. Discussion

siRNA therapy is a promising strategy for the treatment of cancer [22,23]. Over-expression of VEGF can facilitate tumor growth due to neovascularization. Consequently, down-regulation of VEGF expression is an emerging strategy for cancer therapy. However, gene silencing relies on delivering siRNA into target cells, and hence an effective and safe delivery vector is considered as the key to successful therapy. In this study, a strategy of combining polymer with lipids in nanoparticles is developed and the nanoparticles are prepared by microfluidic systems.

PEI is a commonly used cationic polymer for nucleic acid delivery. Considering the cytotoxicity of PEI with the molecular weight of 25 KDa, we used PEI-800 in this study. Firstly, we synthesized a series of P/LNPs and fixed the composition of lipids to DODMA, egg PC, Chol, DSPE-PEG2000 and PEI at the molar ratio of 40/19/35/1/5. In order to produce homogeneous particles with a smaller size for better gene transfection, microfluidic chips were used, as shown in the graph in the Graphic Abstract. 

First, the influences of the flow rate and sonication on synthesis of P/LNPs were investigated. As the flow rate increased, the average size of vesicles showed a decreasing trend, and sonication reduced in size as well. However, neither high flow rate nor sonication narrowed down the size distribution. Therefore, we synthesized P/LNPs at the flow rate of 0.8 mL/min plus sonication in this study. Data from Figure 1 also showed that homogenous P/LNPs with smaller size can be produced by the MF method rather than the BM method.

Secondly, gel retardation assay was used to evaluate siRNA binding property and serum stability of the nanoparticles. The results demonstrated that P/LNPs not only complexed VEGF siRNAs efficiently at the mass ratio of 10:1, but also protected siRNA from serum degradation (Figure 2). Furthermore, MTT assay revealed that no toxicity was observed in A549 and MCF-7 cells when incubated with P/LNPs at the transfection concentration (Figure 3). These data illustrated that P/LNPs were a safe and stable carrier for further study.

Finally, in order to investigate whether P/LNPs can successfully deliver VEGF siRNA into cells, we evaluated siRNA transfection efficiency by confocal microscopy, flow experiment and western blot. In confocal microscopy, bright fluorescence was observed in A549 and in MCF-7 cells, which were incubated with Cy3-siRNA loaded P/LNPs. P/LNPs-MF2 nanoparticles treated cells showed the brightest fluorescence among three P/LNPs groups (Figure 5). Besides, in flow cytometry experiment, fluorescence peaks of cells treated with P/LNPs moved in the right direction compared to the free siRNA group and untreated control group, and the fluorescence peak of P/LNPs was furthest to the right in the figure (Figure 4), which is consistent with the results of confocal data, suggesting that P/LNPs-MF2 delivered more siRNA into cells. Western blot analysis (Figure 6) also showed that P/LNPs-MF2 demonstrated the biggest down-regulation effect of VEGF gene expression in protein level in A549 cells and MCF-7 cells. The improved transfection efficiency of P/LNPs-MF2 over VEGF could be due to the following reasons: (i) P/LNPs with smaller size and more uniform structures can be produced by MF method [13]; (ii) siRNA-loaded LNPs formed by MF methods have electron-dense cores and higher siRNA encapsulation efficiency [16,24]. Further investigation of the mechanism is warranted.

## 4. Materials and Methods

### 4.1. Materials

1,2-Dioleyloxy-*N*,*N*-dimethyl-3-aminopropane (DODMA) was purchased from Corden Pharma Switzerland LLC (Cambridge, MA, USA), l-α-phosphatidylcholine (egg PC), cholesterol (Chol) and 1,2-distearoyl-sn-glycero-3-phosphoethanolamine-*N*-[methoxy(polyethylene glycol)-2000] (DSPE-PEG2000) were obtained from Avanti Polar Lipids Inc. (Alabaster, AL, USA). DSPE-poly(ethylene glycol)-maleimide (DSPE-PEG-MAL-2000) were obtained from Nanosoft Polymers (Lewisville, NC, USA). Branched PEI with molecular weight of 800 Da (PEI 800) and 3-(4,5-dimethylthiazol-2yl)-2,5-diphenyltetrazolium bromide (MTT) was supplied from Sigma-Aldrich (St. Louis, MO, USA). VEGF siRNA (Sense sequence: GGAGUACCCUGAUGAGAUCdTdT-3′; antisense sequence: 5′-GAUCUCAUCAGGGUACUCCdTdT-3′) were provided by Guangzhou RiboBio Co., Ltd. (Guangzhou, China). All cell culture media and supplies were purchased from Gibco (Bethesda, MD, USA).

### 4.2. Preparation of P/LNPs

P/LNPs were prepared by both MF and bulk mixing (BM) methods [17]. DODMA, EggPC, Chol, DSPE-mPEG2000 and PEI-800 at a molar ratio of 40/19/35/1/5 dissolved in absolute ethanol (EtOH) in a lipid/ polymer solution. VEGF siRNA dispersed in HEPES (20 mM HEPES, pH = 4) was prepared as siRNA solution. For BM method, the lipid/polymer solution was loaded into a 1 mL glass syringe and quickly injected into siRNA solution to form the P/LNPs followed by sonication and dialysis against HEPES buffer (20 mM HEPES, pH = 7.4) by a MWCO 10 kDa Float-ALyzer to remove residual ethanol.

For the MF method, two microfluidic chips were used to produce P/LNPs as shown in Figure 2. The lipid/polymer solution was loaded into a 1 mL glass syringe, meanwhile, siRNA solution was loaded into 3 mL glass syringes. All these syringes were connected to the microfluidic chip with tubing and the formulation process of P/LNPs was controlled by syringe pumps. The lipid/PEI stream was hydrodynamically focused by siRNA stream at the crossing point A and the resulting P/LNPs was collected at the outlet port, followed by sonication as described above and dialysis against HEPES buffer (20 mM HEPES, pH = 7.4) to remove residual ethanol using a MWCO 30 kDa Float-A-Lyzer. Finally, the product was sterilized by using a 0.22 μm filter.

### 4.3. Cell Culture

MCF-7 cells were grown in DMEM culture medium supplemented with 10% FBS and 1% *v*/*v* antibiotic/antimycotic, and A549 cells were cultured in RPM1640 supplemented with 10% FBS and 1% *v*/*v* antibiotic/antimycotic, at 37 °C in a humidified atmosphere containing 5% CO_2_.

### 4.4. Zeta Potential and Particle Size Measurements

The particle size and zeta potentials were measured by a NICOMP 380 ZLS Particle Sizing Systems (Santa Barbara, CA, USA) at 25 °C. All Results are given as mean ± standard deviation of three replicates.

### 4.5. Agarose Gel Electrophoresis

A gel retardation assay was carried out to evaluate nanoparticle affinity to siRNA and serum stability. For siRNA binding study, P/LNPs were mixed with siRNA at mass ratio of 10. After 30 min, the samples with 6 × loading buffer were loaded in to 1% agarose gel. The agarose gel electrophoresis (1%, *w*/*v*) containing 0.3% ethidium bromide (EB) was run at 130 V for 15 min in 1 × Tris-Acetate-EDTA (TAE). The gel was visualized and photographed under UV illumination. For serum stability assay, P/LNPs-siRNA were incubated with 50% FBS at 37 °C for 12 h. At different time intervals (0, 1, 2, 4, 8 and 12 h), samples were collected and electrophoresis was carried out as described above. Nake siRNA and FBS treated siRNA were used as control.

### 4.6. Cytotoxicity Assay

Cells were seeded in a 96-well plate at a density of 6000 cells/well and cultured at a standard condition (37 °C, 5% CO_2_). The P/LNPs were diluted in culture medium and added to the cells. At 4 h post-transfection, the medium was replaced by fresh medium and cells were cultured for another 48 h. Then, 20 μL MTT reagent was added to each well and the plate was incubated at 37 °C for 4 h. After that, the medium was removed and 150 μL DMSO was added to dissolve the crystals formed. The cell viability was measured at 490 nm wavelength on a BioTek Synergy 4 Hybrid Microplate Reader (BioTek Instruments, Inc., Winooski, VT, USA).

### 4.7. Flow Cytometric Analysis

Cells were seeded in a 24-well plate at a density of 5 × 10^4^ cells/well and cultivated for 24 h at 37 °C, 5% CO_2_. FAM-siRNA loaded P/LNPs were added to the cells. At 4 h post transfection, the cells were rinsed with cold PBS (pH = 7.4), harvested, fixed in 4% paraformaldehyde solution, and fluorescence intensity was detected on EPICS XL flow cytometer (Beckman Coulter Inc., Brea, CA, USA).

### 4.8. Confocal Microscopy Studies

Cells were seeded in 24-well plate at a density of 3 × 10^4^ cells/well and cultured for 24 h. Cy3-labeled siRNA-loaded P/LNPs were added into each well. At 4 h post transfection, cells were washed 3 times with cold PBS, and fixed with 4% paraformaldehyde solution for 15 min. Then DAPI were used to stain cell nuclei, finally, and images were obtained by Zeiss 710 LSMNLO Confocal Microscope (Carl Zeiss, Oberkochen, Germany).

### 4.9. Western Blot Analysis

Western blot was carried out to evaluate the down regulation of VEGF protein. Briefly, the cells were seeded in 6-well plate at a density of 1 × 10^5^ cells/well and treated with P/LNPs for 48 h. The cells were harvested and protein content was determined by Bicinchoninic Acid (BCA) Protein Assay kit (DingGuo, Beijing, China). After the SDS-PAGE and incubation with the primary and secondary antibodies, immunostaining was performed via the enhanced chemiluminescence (ECL) kit (GE Healthcare, Buckinghamshire, UK), and then submitted to analysis on a Biospectrum 600 Imaging System (UVP, Upland, CA, USA ) to determined gene silencing.

## 5. Conclusions

In summary, we demonstrated that homogeneous P/LNPs with smaller size can be obtained by the MF method, and P/LNPs significantly enhanced the siRNA transfection and gene silencing efficiency with low cytotoxicity in vitro compared to previously reported formulations, especially P/LNPs-MF2. This suggests that P/LNPs-MF2 could be a safe and efficient carrier for siRNA delivery. Furthermore, the strategy of combining cationic polymer with pH-sensitive lipids can be a promising platform for developing effective nanoparticles.

## References

[1] Golkar N., Samani S.M., Tamaddon A.M. (2016). Modulated cellular delivery of anti-VEGF siRNA (bevasiranib) by incorporating supramolecular assemblies of hydrophobically modified polyamidoamine dendrimer in stealth liposomes. Int. J. Pharm..

[2] Kim H.A., Nam K., Kim S.W. (2014). Tumor targeting RGD conjugated bio-reducible polymer for VEGF siRNA expressing plasmid delivery. Biomaterials.

[3] Yin H., Kanasty R.L., Eltoukhy A.A., Vegas A.J., Dorkin J.R., Anderson D.G. (2014). Non-viral vectors for gene-based therapy. Nat. Rev. Genet..

[4] Liu X.X., Rocchi P., Peng L. (2012). Dendrimers as non-viral vectors for siRNA delivery. New J. Chem..

[5] Wang F.J., Yu L., Monopoli M.P., Sandin P., Mahon E., Salvati A., Dawson K.A. (2013). The biomolecular corona is retained during nanoparticle uptake and protects the cells from the damage induced by cationic nanoparticles until degraded in the lysosomes. Nanomed. Nanotechnol. Biol. Med..

[6] Yim H., Park S.J., Bae Y.H., Na K. (2013). Biodegradable cationic nanoparticles loaded with an anticancer drug for deep penetration of heterogeneous tumours. Biomaterials.

[7] Neuberg P., Kichler A. (2014). Recent Developments in Nucleic Acid Delivery with Polyethylenimines. Adv. Genet..

[8] Tripathi S.K., Singh V.P., Gupta K.C., Kumar P. (2013). Hydrophobic and membrane permeable polyethylenimine nanoparticles efficiently deliver nucleic acids in vitro and in vivo. J. Mater. Chem. B.

[9] Xie J., Teng L.S., Yang Z.G., Zhou C.G., Liu Y., Yung B.C., Lee R.J. (2013). A Polyethylenimine-Linoleic Acid Conjugate for Antisense Oligonucleotide Delivery. Biomed. Res. Int..

[10] Guo Z.H., Li Y.J., Fu Y.G., Guo T.Q., Li X., Yang S., Xie J. (2014). Enhanced Antisense Oligonucleotide Delivery Using Cationic Liposomes Incorporating Fatty Acid-Modified Polyethylenimine. Curr. Pharm. Biotechnol..

[11] Yang S., Lee R.J., Yang X.W., Zheng B., Xie J., Meng L.J., Liu Y., Teng L.S. (2015). A novel reduction-sensitive modified polyethylenimine oligonucleotide vector. Int. J. Pharm..

[12] Farokhzad O.C., Khademhosseini A., Yon S.Y., Hermann A., Cheng J.J., Chin C., Kiselyuk A., Teply B., Eng G., Langer R. (2005). Microfluidic system for studying nanoparticles and microparticles the interaction of with cells. Anal. Chem..

[13] Valencia P.M., Farokhzad O.C., Karnik R., Langer R. (2012). Microfluidic technologies for accelerating the clinical translation of nanoparticles. Nat. Nanotechnol..

[14] Hood R.R., DeVoe D.L. (2015). High-Throughput Continuous Flow Production of Nanoscale Liposomes by Microfluidic Vertical Flow Focusing. Small.

[15] Belliveau N.M., Huft J., Lin P.J., Chen S., Leung A.K., Leaver T.J., Wild A.W., Lee J.B., Taylor R.J., Tam Y.K. (2012). Microfluidic Synthesis of Highly Potent Limit-size Lipid Nanoparticles for in Vivo Delivery of siRNA. Mol. Ther. Nucl. Acids.

[16] Balbino T.A., Aoki N.T., Gasperini A.A.M., Oliveira C.L.P., Azzoni A.R., Cavalcanti L.P., de la Torre L.G. (2013). Continuous flow production of cationic liposomes at high lipid concentration in microfluidic devices for gene delivery applications. Chem. Eng. J..

[17] Yang Z., Yu B., Zhu J., Huang X., Xie J., Xu S., Yang X., Wang X., Yung B.C., Lee L.J. (2014). A microfluidic method to synthesize transferrin-lipid nanoparticles loaded with siRNA LOR-1284 for therapy of acute myeloid leukemia. Nanoscale.

[18] Karnik R., Gu F., Basto P., Cannizzaro C., Dean L., Kyei-Manu W., Langer R., Farokhzad O.C. (2008). Microfluidic platform for controlled synthesis of polymeric nanoparticles. Nano Lett..

[19] Valencia P.M., Basto P.A., Zhang L.F., Rhee M., Langer R., Farokhzad O.C., Karnik R. (2010). Single-Step Assembly of Homogenous Lipid–Polymeric and Lipid–Quantum Dot Nanoparticles Enabled by Microfluidic Rapid Mixing. ACS Nano.

[20] Tan S., Li X., Guo Y., Zhang Z. (2013). Lipid-enveloped hybrid nanoparticles for drug delivery. Nanoscale.

[21] Dehaini D., Fang R.H., Luk B.T., Pang Z., Hu C.M., Kroll A.V., Yu C.L., Gao W., Zhang L. (2016). Ultra-small lipid-polymer hybrid nanoparticles for tumor-penetrating drug delivery. Nanoscale.

[22] Ozpolat B., Sood A.K., Lopez-Berestein G. (2014). Liposomal siRNA nanocarriers for cancer therapy. Adv. Drug Deliv. Rev..

[23] Shen H., Sun T., Ferrari M. (2012). Nanovector delivery of siRNA for cancer therapy. Cancer Gene Ther..

[24] Leung A.K., Hafez I.M., Baoukina S., Belliveau N.M., Zhigaltsev I.V., Afshinmanesh E., Tieleman D.P., Hansen C.L., Hope M.J., Cullis P.R. (2012). Lipid Nanoparticles Containing siRNA Synthesized by Microfluidic Mixing Exhibit an Electron-Dense Nanostructured Core. J. Phys. Chem. C Nanomater. Interfaces.

